# The ischaemic preconditioning paradox and its implications for islet isolation from heart-beating and non heart-beating donors

**DOI:** 10.1038/s41598-022-23862-x

**Published:** 2022-11-11

**Authors:** Daniel Brandhorst, Heide Brandhorst, Samuel Acreman, Paul R. V. Johnson

**Affiliations:** Research Group for Islet Transplantation, Nuffield Department of Surgical Sciences, John Radcliffe Hospital, University of Oxford, Oxford, OX3 9DU UK

**Keywords:** Regenerative medicine, Biologics, Cell therapies, Molecular medicine

## Abstract

The impact of ischaemia can severely damage procured donor organs for transplantation. The pancreas, and pancreatic islets in particular, is one of the most sensitive tissues towards hypoxia. The present study was aimed to assess the effect of hypoxic preconditioning (HP) performed ex-vivo in islets isolated from heart-beating donor (HBD) and non heart-beating donor (NHBD) rats. After HP purified islets were cultured for 24 h in hypoxia followed by islet characterisation. Post-culture islet yields were significantly lower in sham-treated NHBD than in HBD. This difference was reduced when NHBD islets were preconditioned. Similar results were observed regarding viability, apoptosis and in vitro function. Reactive oxygen species generation after hypoxic culture was significantly enhanced in sham-treated NHBD than in HBD islets. Again, this difference could be diminished through HP. qRT-PCR revealed that HP decreases pro-apoptotic genes but increases HIF-1 and VEGF. However, the extent of reduction and augmentation was always substantially higher in preconditioned NHBD than in HBD islets. Our findings indicate a lower benefit of HBD islets from HP than NHBD islets. The ischaemic preconditioning paradox suggests that HP should be primarily applied to islets from marginal donors. This observation needs evaluation in human islets.

## Introduction

In order to overcome the extreme disbalance between organ donation rates and the continuously rising waiting lists of patients awaiting transplantation^1^, the worldwide utilisation of donors after cardiac death (DCD) is continuously growing. In U.K., the DCD donation rate increased 17-fold from 2000 to 2020^2^. In Europe, the utilisation rate of DCD over total deceased donors was in average 12.7% as calculated for 2008–2016^3^. However, the most recent U.K. transplant activity report from 2021, covering 12 months of time, noted that ten islet preparations could be successfully isolated from donors after brain death (DBD) whilst none could be transplanted from the pancreas of DCD^4^. One of the most relevant impacts that can severely injure procured organs are primarily normothermic and cold ischaemia followed by ischaemia/reperfusion injury after implantation^5^. The pancreas, and particularly its endocrine compartment, is one of the most sensitive tissues with respect to ischaemia or hypoxia. Islets of Langerhans represent only a minute fraction of 1–2% of the pancreas but receive approximately 15–20% of the entire blood flow^6,7^. Any interruption of the blood supply or oxygen delivery has severe and immediate consequences for the metabolic and morphologic integrity of islets^8–10^. Beside this specific sensitivity, islets also share the low protective antioxidant capacity of the brain, the organ that is most sensitive to the lack of oxygen^11–14^. Recent studies favour advanced perfusion techniques to protect retrieved pancreases from ischaemia and reperfusion injury^15^. In contrast to these complex techniques, which require continuous observation and manipulation during organ storage and shipment, ischaemic preconditioning is a simple procedure that can be applied during organ retrieval prior to organ transportation^16^. The term ischaemic preconditioning refers to short alternating periods of ischaemia and reperfusion of organs in order to induce protection against subsequent prolonged periods of ischaemia during procurement and/or shipment^17^. Amongst different organs which have been subjected to ischaemic preconditioning, the liver is surely the tissue most investigated and that was the first organ to be translated from the laboratory to the clinical setting^18^. In contrast, the number of studies about ischaemic preconditioning of the pancreas is very low when compared with studies in the liver, heart or kidney^19,20^. This applies particularly when considering studies that include islet isolation. To the best of our knowledge, only two experimental studies have been undertaken so far to assess the effect of ischaemic preconditioning of the pancreas prior to islet isolation^21,22^. Both studies demonstrated a beneficial effect on islet isolation outcome and increased islet quality after prolonged ischaemia, but to date these have not led to any studies in human pancreases.

To circumvent the logistic difficulties of implementing ischaemic preconditioning into routine pancreas procurement for subsequent islet isolation^23,24^, the aim of the present study has been to assess the effect of ischaemic preconditioning on already isolated islets using the rat model. In contrast to a previously performed approach to investigate the effect of hypoxia on the secretory response of preconditioned mouse islets^25^ we implemented two different categories of donors, termed heart-beating donors (HBD) and non heart-beating donors (NHBD), in the experiments. Although out of date^26^, we use this terminology because it fits better to the organ procurement in rats compared with the currently used standard terms DBD and DCD for human organ donors. Because our experimental design included temporary exposure of islets to a hypoxic atmosphere rather than interrupting the blood supply for pancreatic tissue we also altered the terminology from ischaemic to hypoxic preconditioning (HP).

## Results

### Islet yield and morphological integrity

**Figure 1:**
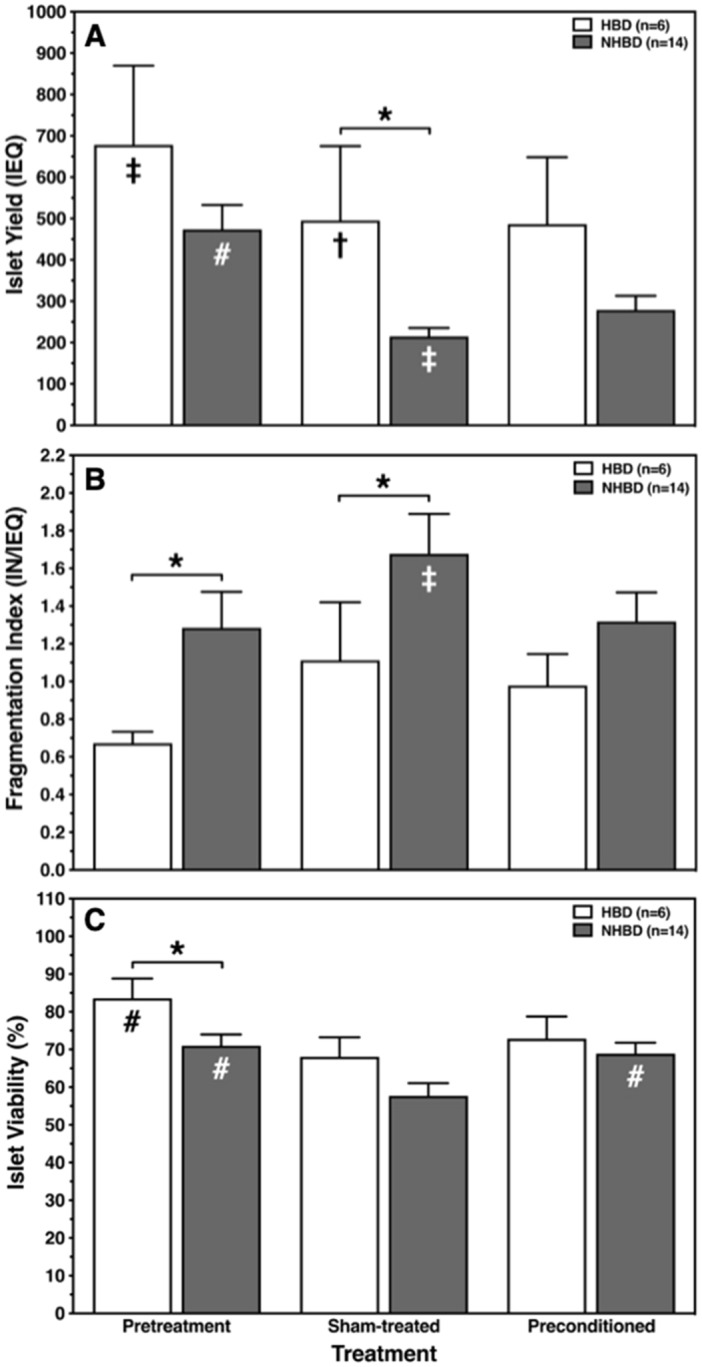
Ex-vivo hypoxic preconditioning improves morphological integrity of ischemically predamaged rat islets. Islets were isolated from heart-beating (HBD, n = 6, white bars) and non heart-beating donors (NHBD, n = 14, grey bars). Islet yield (**A**), fragmentation index (**B**) and viability (**C**) were assessed immediately post isolation (pretreatment) and after sham-treatment or preconditioning followed by 24 h-culture of preconditioned or sham-treated islets in hypoxic atmosphere, respectively. *p < 0.05 for HBD versus NHBD. Symbols inside bars indicate †*p* < 0.05 versus corresponding pretreatment; ‡*p* < 0.01 versus corresponding preconditioning; #*p* < 0.001 versus corresponding sham-treatment.

The outcome of islet isolation from the pancreas of HBD and NHBD is shown in Fig. 1. As demonstrated in Fig. 1A, the pretreatment, i.e. freshly isolated islet yield of HBD was similar compared with NHBD. The loss of sham-treated islets after culture in hypoxic atmosphere was more pronounced in NHBD than in HBD but could be significantly ameliorated when NHBD islets had been preconditioned (*p* < 0.01 vs. sham-treated). After culture in hypoxic atmosphere the difference between HBD and NHBD became significant in sham-treated islets (*p* < 0.05) but not in islets preconditioned with hypoxia. Reduction of islet yield correlated with increased islet fragmentation as expressed by the fragmentation index (IN/IEQ) (Fig. 1B). Freshly isolated and sham-treated islets from NHBD were characterised by a significantly higher fragmentation index compared with HBD islets (*p* < 0.05). The difference between the fragmentation index of HBD and NHBD could be reduced when NHBD islets had been preconditioned by hypoxia.

Membrane integrity of pretreatment islets as measured by the FDA-PI assay was significantly higher in HBD than in NHBD (*p* < 0.05) (Fig. 1C). After culture in hypoxic atmosphere, viability was significantly lowest in sham-treated NHBD islets when compared with freshly isolated and preconditioned islets (*p* < 0.001). However, when islets from NHBD had been preconditioned, viability increased to the level of pretreatment islets.

### Islet mitochondrial activity

**Figure 2:**
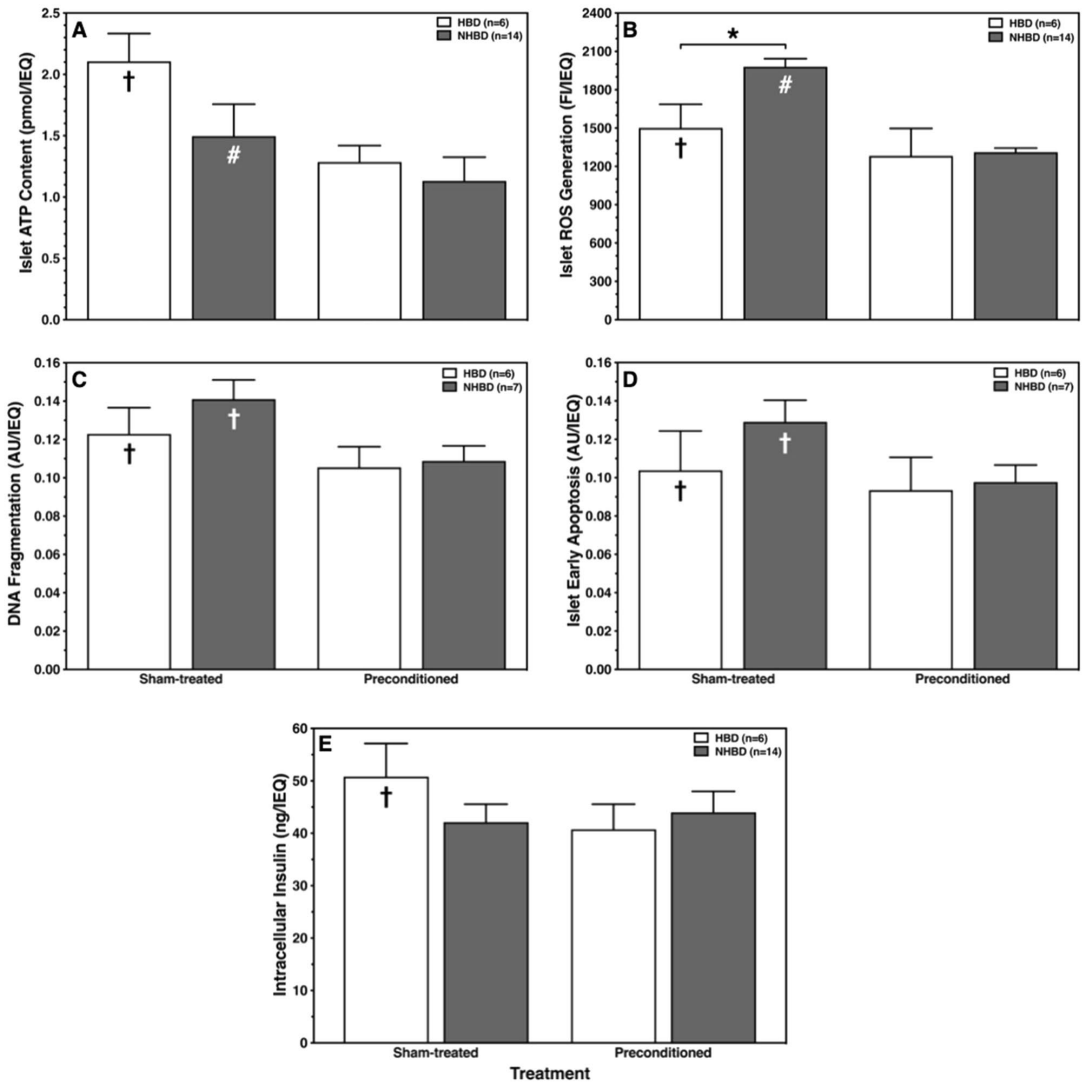
Ex-vivo hypoxic preconditioning stabilises ATP production and reduces ROS generation of ischemically predamaged rat islets. Islets were isolated from heart-beating (HBD, white bars) and non heart-beating donors (NHBD, grey bars). (**A**) ATP content, (**B**) ROS production, (**C**) DNA fragmentation, (**D**) early apoptosis and (**E**) intracellular insulin content were assessed after sham-treatment or preconditioning followed by 24 h-culture of sham-treated or preconditioned islets in hypoxic atmosphere, respectively. *p < 0.05 for HBD versus NHBD. Symbols inside bars indicate †*p* < 0.05, #*p* < 0.001 versus corresponding preconditioning.

A trend toward a lower ATP content was observed in sham-treated islets isolated from NHBD when compared with corresponding HBD islets (NS) (Fig. 2A). HP additionally reduced the islet ATP content in both HBD (*p* < 0.05 vs. sham-treated) as well as in NHBD (*p* < 0.001).

An inverse but fitting pattern was observed for the ROS production of islets (Fig. 2B). The generation of ROS after culture in hypoxic atmosphere was substantially lower in sham-treated HBD islets compared with corresponding NHBD islets (*p* < 0.05). In contrast, HP diminished this difference between donor categories and significantly lowered the ROS production in HBD islets (*p* < 0.05 vs. sham-treated) and particularly in NHBD islets (*p* < 0.001) to a nearly identical level (Fig. 2B).

DNA fragmentation clearly correlated with ROS production as shown in Fig. 2C. The DNA fragmentation in sham-treated islets was identical in NHBD and HBD but was significantly reduced and equalised by HP in both donor categories (*p* < 0.05 vs. sham-treated).

DNA fragmentation was paralleled by signs of early apoptosis as determined by Annexin-V staining (Fig. 2D). In any donor category this variable was noted to be significantly higher in sham-treated than in preconditioned islets (*p* < 0.05) where a similar level of Annexin-V expression was measured in HBD and NHBD islets.

No differences were found between donor categories with respect to the intracellular storage of insulin (Fig. 2E). However, when HBD islets were subjected to HP a significantly decreased insulin content was measured (*p* < 0.05).

### Islet gene expression

The findings in terms of DNA fragmentation correlated with the gene expression of the DNA damage-inducible transcript-3 CHOP which was significantly reduced in preconditioned islets isolated from HBD (*p* < 0.01 vs. sham-treated) or from NHBD (*p* < 0.001 vs. sham-treated). As shown in Table 1, the reduction was significantly higher in NHBD than in HBD islets (*p* < 0.01). A similar observation was made for Bax, another pro-apoptotic key marker, which was significantly decreased after preconditioning of islets from HBD (*p* < 0.01 vs. sham-treated). However, when islets from NHBD were preconditioned a massive reduction of Bax mRNA expression was noted (*p* < 0.001 vs. sham-treated).

**Table 1:** Effect of hypoxic preconditioning on mRNA expression of cultured rat islets isolated from heart-beating (HBD, n = 7) or non heart-beating (NHBD, n = 7) donors. mRNA expression is displayed as n-fold change compared with sham-treated islets and was measured by qRT-PCR after 24 h of culture in hypoxia.

mRNA expression	Donor category		*p*-value
(n-fold)	HBD	NHBD	
CHOP	0.676 ± 0.033‡	0.418 ± 0.082#	< 0.01
Bax	0.700 ± 0.053‡	0.190 ± 0.104#	< 0.01
HIF-1α	1.244 ± 0.169‡	3.402 ± 0.638#	< 0.01
VEGF-A	1.836 ± 0.591	4.154 ± 0.551#	< 0.05
HO-1	0.826 ± 0.124	0.333 ± 0.067#	< 0.01

^†^*p* < 0.05, ‡*p* < 0.01, #*p* < 0.001 versus corresponding sham-treated.

In addition to pro-apoptotic genes, protective genes like HIF-1α or VEGF-A, were also upregulated in preconditioned islets. This upregulation was particularly pronounced in islets from NHBD (*p* < 0.001 vs. sham-treated) whilst HBD islets showed a significant increase only for HIF-1α (*p* < 0.01 vs. sham-treated) but not for VEGF-A (NS). Again, the difference between the donor categories was significant for HIF-1α (*p* < 0.01) as well as for VEGF-A (*p* < 0.05).

The mRNA expression of HO-1 followed more or less the mRNA expression pattern of CHOP and Bax. HO-1 was only marginally decreased in HBD islets (NS vs. sham-treated) whilst being significantly downregulated in islets from NHBD (*p* < 0.001 vs. sham-treated).

### Islet secretory capacity

**Figure 3:**
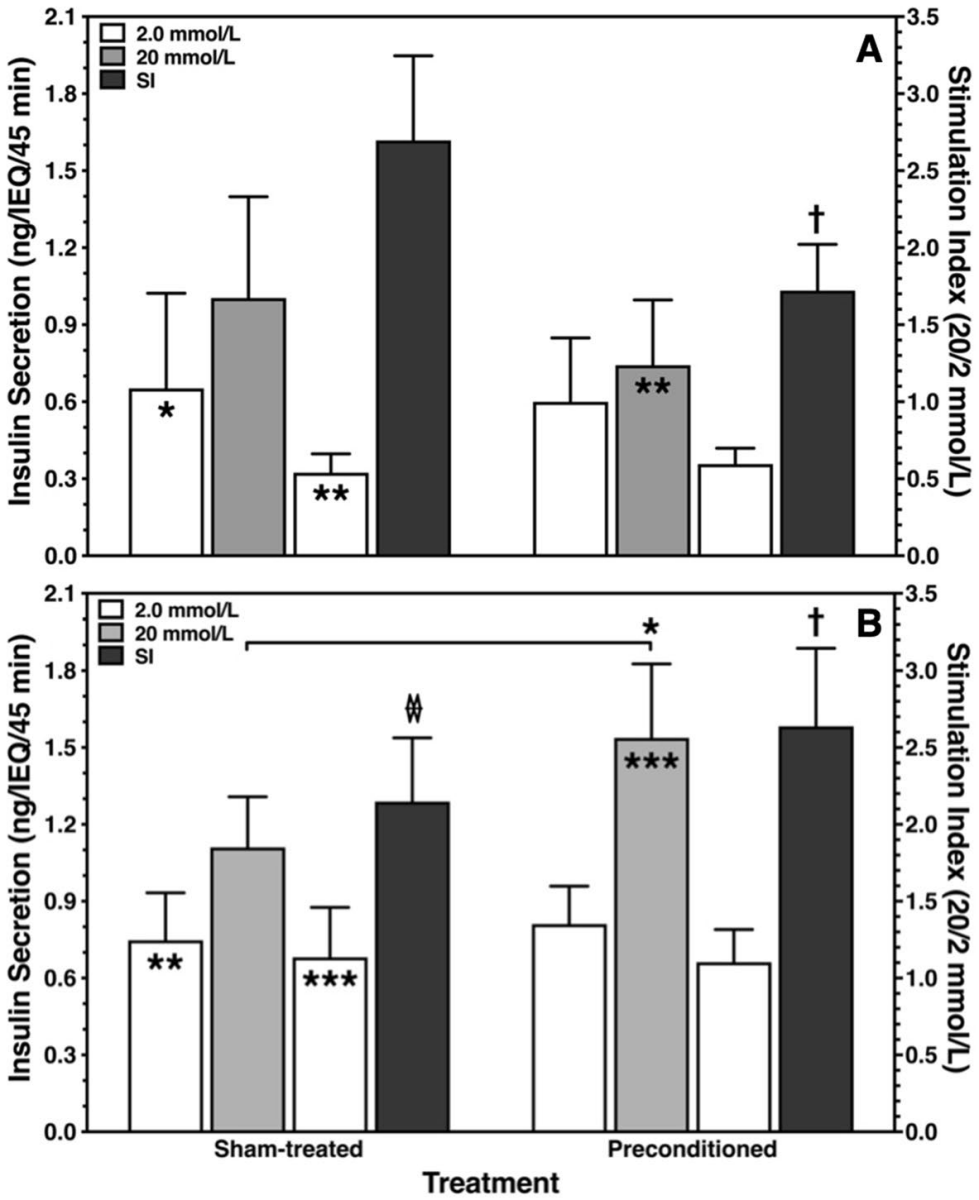
Ex-vivo hypoxic preconditioning enhances secretory capacity of ischemically predamaged rat islets. Islets were isolated from (**A**) heart-beating (n = 6) and (**B**) non heart-beating donors (n = 14). Glucose challenge of sham-treated or preconditioned rat islets was performed after 24 h of culture in hypoxic atmosphere. The ratio of stimulated insulin release (20 mmol/L, grey bars) over the mean of the two basal periods (2 mmol/L, white bars) of 10 rat islets is expressed as glucose stimulation index (SI, black bars). Symbols inside bars indicate *p < 0.05, **p < 0.01, ***p < 0.001 for 2.0 versus 20 mmol/L of glucose. †*p* < 0.05 for SI of preconditioning versus sham-treatment. (**B**) *p < 0.05 for sham-treatment versus preconditioning as indicated. *p* < 0.05 for SI of sham-treated NHBD versus sham-treated HBD by Mann–Whitney test.

The insulin secretory capacity of sham-treated and preconditioned islets isolated from HBD (Fig. 3A) or NHBD (Fig. 3B) was assessed by means of sequential static glucose incubation after 24 h of culture in hypoxic atmosphere. In HBD islets, the SI after HP was significantly lower compared with sham-treatment (*p* < 0.05). Remarkably, an opposite observation was made for islets isolated from NHBD (Fig. 3B). In this donor category, preconditioned islets were characterised by a significantly higher stimulated insulin release (*p* < 0.05 vs. sham-treated) resulting in a larger SI compared with sham-treated islets (*p* < 0.05). As a result, the SI of sham-treated islets isolated from NHBD was significantly lower compared with corresponding islets from HBD (*p* < 0.05 by Mann–Whitney test) which was not the case when preconditioned islets of both donor categories were compared. Nevertheless, all experimental groups of HBD and NHBD islets responded positively to a glucose challenge even after 24 h of culture in hypoxia.

### Islet overall survival

**Figure 4:**
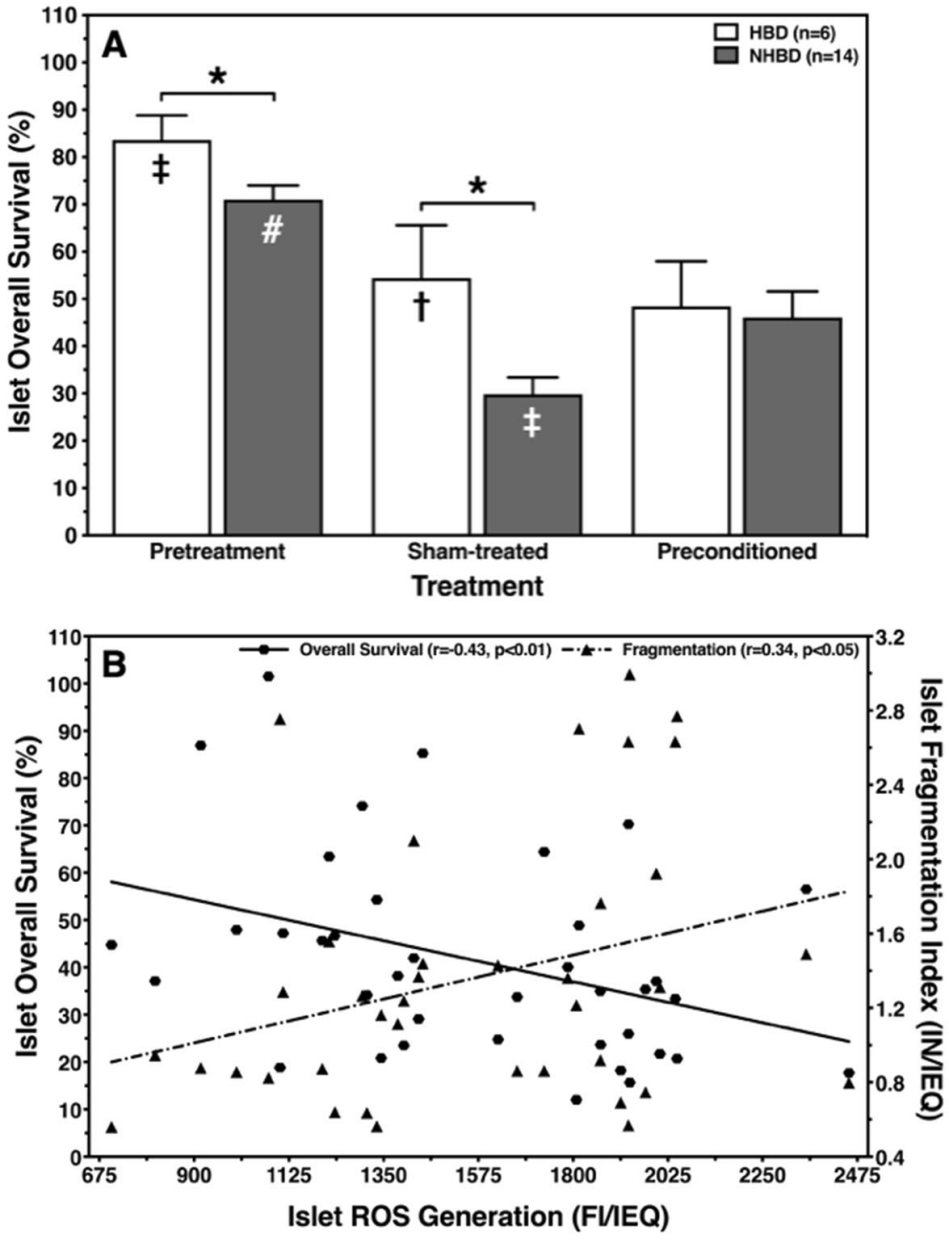
Ex-vivo hypoxic preconditioning increases overall survival of ischemically predamaged rat islets via ROS production. Islets were isolated from heart-beating (HBD, white bars, n = 6) and non heart-beating donors (NHBD, grey bars, n = 14). (**A**) Overall survival was assessed after preconditioning or sham-treatment followed by 24 h-culture of preconditioned or sham-treated islets in hypoxic atmosphere, respectively. (**B**) Effect of ROS production in sham-treated and preconditioned rat islets during culture in hypoxic atmosphere on postculture overall survival and fragmentation. The correlation coefficient (r) was calculated using Spearman’s rank correlation. *p < 0.05 for HBD versus NHBD. Symbols inside bars indicate †*p* < 0.05, ‡*p* < 0.01 versus corresponding preconditioning; #*p* < 0.001 versus corresponding sham-treatment.

Islet overall survival was calculated as an integrative parameter that can reflect islet potency despite its simplicity in calculation. As shown in Fig. 4A, the initial overall survival of pretreatment islets was significantly higher when isolated from HBD compared with NHBD (*p* < 0.05). Whilst overall survival of HBD islets decreased to a nearly identical level after sham-treatment and preconditioning (*p* < 0.01 vs. pretreatment), the reduction of this parameter was substantially stronger in NHBD islets after sham-treatment (*p* < 0.001 vs. pretreatment; *p* < 0.05 vs. HBD). Nevertheless, the differences between HBD and NHBD islets were minimised when islets from NHBD had been preconditioned.

Correlation analysis revealed that morphological integrity and overall survival of sham-treated and preconditioned islets was significantly affected by the ROS production in islets cultured in hypoxic atmosphere. As demonstrated in Fig. 4B, increasing amounts of intraislet ROS enhanced islet fragmentation (r = 0.34, *p* < 0.05) while islet overall survival inversely correlated with ROS production (r =  − 0.43, *p* < 0.01).

## Discussion

Ischaemia and hypoxia have been identified as a major cause of injury to donor pancreases and are decisive factors for morphological and functional integrity of isolated islets^27,28^. The substantially decreased oxygen supply during organ procurement, isolation procedure, islet culture and transplantation is closely linked to inflammation^29,30^. Despite the lack of oxygen, proinflammatory pathways paradoxically trigger an excessive production of ROS which mediates oxidation of enzymes and structural proteins as well as DNA fragmentation and apoptosis finally resulting in islet dysfunction and cell death^31–34^. In this context, it is important to stress that islets share the low protective antioxidant capacity of the brain, the organ that is most sensitive to the lack of oxygen^11–14^. In addition, beta cells express a high xanthine oxidase activity^35^ which is responsible for the formation of ROS from hypoxanthine as the final degradation product of hypoxia-induced dephosphorylation of ATP^17^. For that reason it is not surprising that several animal models such as mouse, rat, dog and pig have demonstrated that the pancreas and its endocrine compartment is particularly vulnerable towards normothermic ischaemia, resulting in massive reduction in freshly isolated islet yield, associated with a substantially decreased morphological and functional integrity, even when exposed to hypoxia for a short period of time^36–40^.

Ischaemic preconditioning was introduced as a protective method to prevent ischaemia and reperfusion injury in ischaemic organs nearly four decades ago^41^. In order to ameliorate the hypoxia-induced injury of isolated islets exposed to a hypoxic environment we developed a simple procedure for ischaemic preconditioning that can easily be transferred into a clinical setting. This is in contrast to a previous study which used a complex, technically orientated method to precondition mouse islets by means of hypoxia. However, the emphasis of the study from Lo et al. was to assess stimulus-secretion coupling factors in mouse islets^25^.

After 24-h exposure to hypoxic atmosphere a lower yield of islets, characterised by higher fragmentation, decreased viability as well as upregulated makers of apoptosis, was measured in sham-treated islets isolated from HBD and NHBD. In contrast, a significantly higher yield of islets with less fragmentation, increased viability, a lower rate of apoptosis was observed after HP, particularly in preconditioned islets from NHBD. These differences could not be noted to the same extent when different treatment groups were compared in HBD.

In contrast to islets from HBD, NHBD islets are exposed to prolonged normothermic ischaemia impairing the oxidative production of ATP^8^. As discussed in the previous paragraphs, a low ATP content accompanied by the rapid degradation of ATP into hypoxanthine during ischaemia are the main triggers of intraislet ROS production. In agreement with these considerations, we demonstrated that ischaemically predamaged islets are characterised by a decreased ATP content and an increased ROS production. As a consequence, we observed elevated levels of membrane damage as measured by FDA-PI, apoptosis and DNA fragmentation in sham-treated NHBD islets. Although our data suggest that HP seems to contribute to the ATP consumption in treated islets, we noted significantly inhibitory effects on these parameters in preconditioned islets which is consistent with previous observations in rats suggesting that ischaemic preconditioning significantly reduces the oxidative damage of ischaemia-exposed liver tissue^42^. Again, the protective effect of HP on islets was stronger in NHBD than in HBD.

Surprisingly, the HP-induced incremental reduction in ATP production did not seem to interfere with the secretory capacity of islets measured during glucose-stimulated insulin release in-vitro. The data we collected during these experiments demonstrate that the highest SI is obtained with sham-treated islets from HBD representing the experimental group with the presumably lowest ischaemic stress level during organ procurement. This assay also showed that HP decreases the islet insulin response after glucose challenge in HBD to a level which was even lower than the equivalent parameters of sham-treated islets from NHBD. Compared with HBD islets, preconditioning of NHBD islets significantly improved the SI of these islets reaching a nearly identical magnitude as sham-treated islets from HBD.

Despite the long time that has passed since the introduction of ischaemic preconditioning as experimental procedure to protect procured organs, the exact pathways that trigger the increased resistance against hypoxia in different tissues are still not completely understood^43^. One of the most important contributors to protection is surely the inhibitory effect of ischaemic preconditioning on ROS generation as also observed in the liver^42,44^. Beside this mechanism others are speculative and most likely multi-fold because ischaemic preconditioning has been shown to be protective against different impacts such as hypoxic atmosphere, as used in the present approach, but also against a vigorous reaction like the immediate blood-mediated inflammatory reaction known as IBMIR^22^. Recent experiments suggest that the beneficial effects of ischaemic preconditioning may involve the function of Calcium and K^+^-ATP-dependent channels^45,46^.

Another aspect which can be discussed in this context, is that ischaemic preconditioning seems to increase the expression of antioxidants such as superoxide dismutase and glutathione as shown in the rat liver^44^. Another antioxidative compound that is triggered by ischaemic preconditioning in different organs of the rat is haeme oxygenase-1 (HO-1)^47,48^. Surprisingly, our experiments revealed that HP-induced HO-1 gene expression in islets is downregulated rather than upregulated. Numerous experiments have demonstrated that HO-1 is highly protective for beta cells when exposed to cytokines, high glucose concentrations or nitric oxide generation^49–51^. In the present study, HO-1 was substantially downregulated in preconditioned NHBD islets but not in corresponding HBD islets. However, on the first view our findings implement that HP of islets may reduce the protective capacity of islets subjected to certain harmful conditions. On the other hand, HO-1 has been categorised as stress-induced heat shock protein-32 in rat but not in human islets^52–55^. From this point of view HO-1 can be regarded as a rat-specific stress protein that is a component of the universal heat shock response which enable tissue and cells to survive in otherwise lethal situations^56,57^. Its downregulation may therefore reflect a reduced stress level in preconditioned islets rather than a reduction in the protective capacity.

Our hypothesis fits also to the significantly reduced expression of proapoptotic genes such as CHOP and Bax in preconditioned islets paralleled by the decrease of early apoptosis and DNA fragmentation. As noted for morphological integrity and viability of islets, the extent of reduction is again smaller in preconditioned HBD than in NHBD islets.

The same applies to the substantially increased expression of protective genes such as HIF-1α and VEGF-A in the latter experimental group. Since HIF-1α is controlling numerous target genes^58^, it is not surprising that we also detected a massively upregulated expression of VEGF-A mRNA in NHBD islets. Beside its essential role for islet vascularization and functionality^59,60^, VEGF seems to have an islet-protective effect that is independent of revascularisation and contributes to human islet survival under adverse conditions^61^. The finding, that VEGF-A mRNA is only significantly upregulated in preconditioned NHBD islets but not in corresponding HBD islets, suggests that the major stimulus for VEGF-A is associated with the hypoxic situation during organ retrieval in NHBD. The same implication is provided by the mRNA expression of HIF-1α which is significantly higher in preconditioned islets from NHDB compared with HBD.

Another important role of HIF-1α is to limit the mitochondrial ROS production under hypoxic conditions^62^ which is conform with our observation that the protective effect of HP on the integrity of hypoxia-exposed islets is associated with an inhibitory effect on ROS production. In agreement with our findings, several studies have demonstrated that the expression of HIF-1α is a central part of the metabolic response of islets towards hypoxia contributing to islet survival in an otherwise lethal environment^63–65^. In contrast, other attempts suggested that HIF-1α is rather a specific marker for severe hypoxia and is co-expressed in islets with signs of apoptosis^66–68^. In contrast to these studies, the present approach revealed that gene and cellular markers of early and late apoptosis are significantly reduced in preconditioned islets. The data obtained from other models such as the mouse heart or the rat liver are conflicting as well and have not finally answered the question whether HIF-1α is a central component of the ischaemic preconditioning-induced protection^62,69^ or just a tissue marker of hypoxia^70^. The latter conclusion can also be drawn from experiments with rat islets subjected to remote ischaemic preconditioning^22^.

In the context of hypoxic stress induction of it has to be realised that pancreas digestion itself is associated with the detrimental impact of anoxia, high osmolarity and high enzyme activity^71^. It can be speculated whether the low presence of oxygen during pancreas dissociation may represent a kind of hypoxic preconditioning taking place prior to the actual preconditioning procedure and it can be asked whether a duplicate preconditioning procedure may enhance or reduce the protective effect of preconditioning. However, whether this question is relevant for the outcome of the present study might be denied since the isolation isolation conditions were identical for both donor categories. In addition, the conditions as listed above are inherent variables of the isolation procedure and are difficult to avoid using the current methods for islet isolation.

The finding that HP has not the same protective effect in HBD compared with NHBD is additionally confirmed by islet overall survival in the present study. This integrative parameter additionally demonstrates that HP increases the overall survival of NHBD islet exposed to culture in a hypoxic atmosphere whilst islets from HBD do not significantly benefit from this treatment. This finding is in agreement with the previously described ischaemic preconditioning paradox that reflects the unexpected increase in reperfusion injury after treating the organ of “good” i.e. non-marginal donors^23^. So far and to the best of our knowledge this paradox has not been identified in organs others than the liver^72–74^. It has also not been observed in the only study about remote ischaemic preconditioning prior to intraportal islet transplantation in rats where the results noted for islet yield, morphology, in vitro function and posttransplant outcome do not indicate any significant differences between preconditioned islets and controls^22^. This seems to match to the ischaemic preconditioning paradox as the non-marginal donors in Delaune’s study did not experience any significant normothermic or cold ischaemia.

Whether the ischaemic preconditioning paradox may also be of significance for marginal human pancreas donors such as donors after cardiac death is another controversial issue that has to be discussed. When regarding islet yield as the most relevant determinant for islet transplantation it is implausible that hypoxic preconditioning can improve isolation outcome. Nevertheless, as shown in the present study hypoxic preconditioning benefits islets to acquire more protection against hypoxia which potentially increases graft function and survival after transplantation.

## Conclusion

In summary, to the best of our knowledge the present study is the first one to describe the influence of ischaemic preconditioning paradox on islet isolation. Our data clearly indicate that HP of already isolated islets decreases the intra-islet production of ROS and reduce pro-apoptotic pathways thereby protecting islets from NHBD subsequently exposed to a hypoxic environment, with respect to yield, morphological and functional integrity. In contrast, islets from HBD do not seem to benefit from HP to the same extent as NHBD islets. From these findings we conclude that HP should be mainly applied in islets from marginal donors. Further studies are needed to investigate the effect of HP on islets isolated from human donors.

## Materials and methods

**Figure 5:**
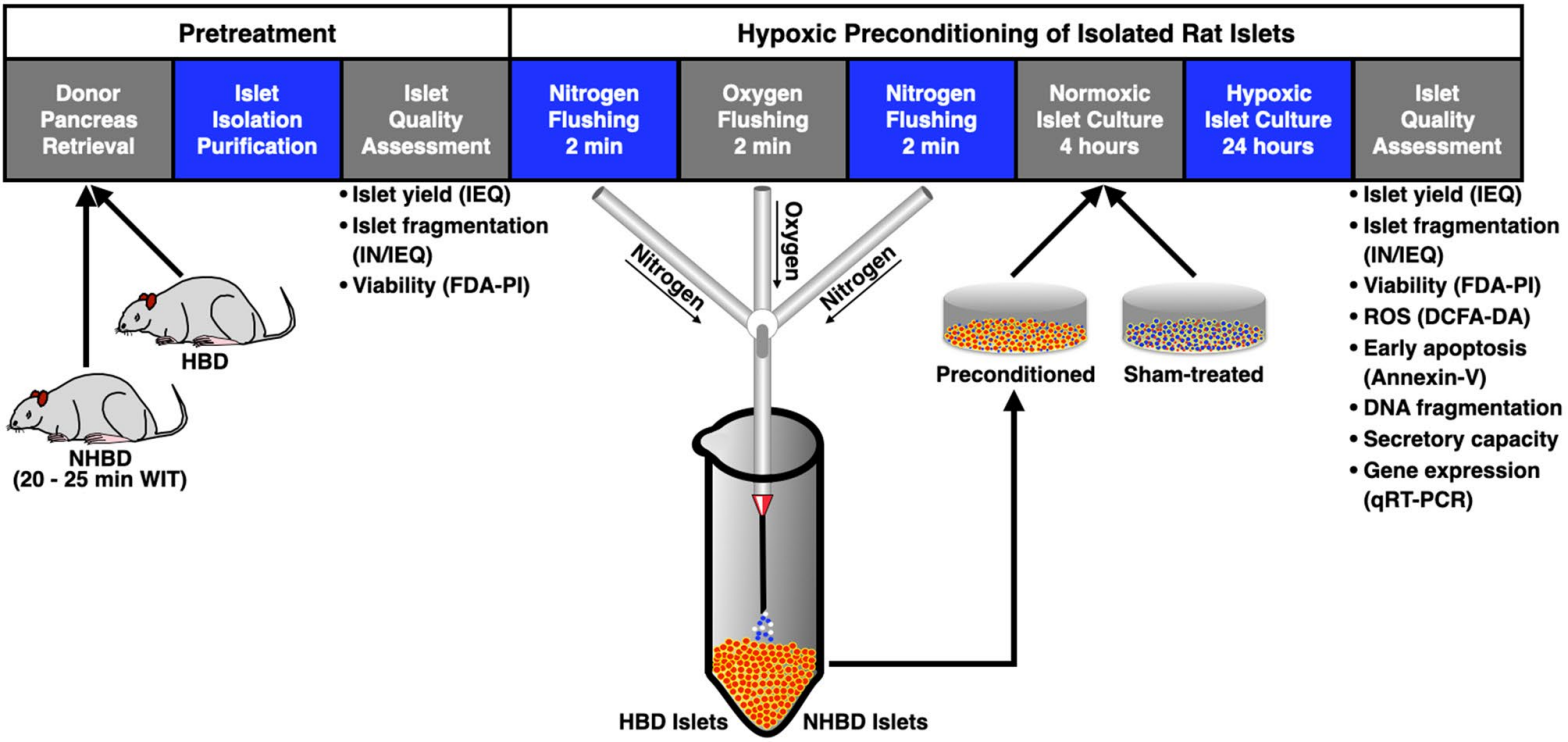
Experimental study design. Rat islets isolated from the pancreas of heart-beating (HBD, n = 7) and non heart-beating donors (NHBD, n = 14) were preconditioning in conical centrifuge tubes using alternating gassing of the islet suspension with nitrogen and oxygen for 2 min prior to recovery of 4 h of normoxic culture and subsequent exposure to hypoxia for 24 h followed by islet quality assessment.

The experimental design of the study is shown in Fig. 5. Islet quality assessment was performed immediately after purification (pretreatment) and post hypoxic culture. All methods were performed in accordance with the relevant guidelines and regulations.

### Pancreas retrieval

All animal studies were carried out in accordance with the ARRIVE guidelines and had been approved by the Committee on Animal Care and Ethical Review of the University of Oxford (PPL 30/3228). Pancreases were obtained from male Lewis rats aged 10–11 weeks and weighing approximately 300–350 g (Harlan Laboratories, Bicester, U.K.).

After induction of anaesthesia with isoflurane, heart-beating donors (HBD) were intraperitoneally injected with 75 mg/kg body weight Ketamine (Biomedical Services, Oxford, U.K.) plus 0.5 mg/kg Medetomidine (Cambridge Biosciences, Cambridge, U.K.) prior to midline incision of the abdomen. After exposure, the pancreas was chilled by pre-cooled gel packs followed by intraductal pancreas distension in situ utilising 10 mL of cold perfusion solution supplemented with 3.1 mM calcium chloride (Lonza, Slough, U.K.), 22 units of 4-phenylazobenzyloxycarbonyl-L-prolyl-L-leucylglycyl-L-prolyl-D-arginine (PZ-U) of collagenase NB-1 (Serva Electrophoresis, Uetersen, Gemany) and 0.5 dimethylcasein units (DMC-U) of neutral protease NB-1 (Serva). Subsequent to distension, the pancreas was mobilised, resected and immersed in cold perfusion solution until stationary digestion at 37 °C. Immediately after pancreas retrieval the donor animal was euthanasied with an intraperitoneal injection of 800 mg/kg BW pentobarbital (Bio-Techne LTD, Abingdon, U.K.).

After induction of anaesthesia with isoflurane non heart-beating donors (NHBD) were killed by cervical dislocation prior to midline incision of the abdomen followed by exposure of the pancreas. After 20–25 min of warm ischaemia, intraductal pancreas distension and pancreas resection were performed in situ as described for HBD.

### Islet isolation

Isolation and purification of rat islets was performed as previously described^75^.

### Ex-vivo hypoxic preconditioning

Subsequent to isolation and purification, rat islets were characterised with respect to yield, morphology and viability and divided into two equal aliquots assigned for preconditioning or for sham-treatment. One of the aliquots was suspended in one mL of serum-free CMRL 1066, supplemented as described above, transferred into a 5 mL-conical centrifuge tube (VWR, Lutterworth, U.K.) and gassed for 2 min with nitrogen supplemented with 5% carbon dioxide followed by a 2 min-period of treatment with carbon dioxide-supplemented oxygen. The gas infusion was performed via a 18G-injection needle (Becton Dickinson, Swindon, U.K.) connected to a silicone tube which had been attached to the appropriate gas regulator (DCGE Services Limited, Banbury, U.K.). The gas pressure and the flow rate was 4 PSI and 15 standard cubic feet per minute, respectively. The seeding density during gassing varied between 170 and 1580 IEQ per mL. After oxygen flushing, islets were finally gassed with nitrogen and transferred into 24- or 12-well plates (Greiner Bio-One, Stonehouse, U.K.) filled with 500 µL or 1000 µL of serum-supplemented CMRL 1066 per well, respectively. Sham-treated islets were handled and treated in the same manner as preconditioned islets apart from the gas application. Prior to 24 h-culture in hypoxic atmosphere (1.5% oxygen) gassed and sham-treated islets were subjected to normoxic culture for 4 h to give islets the opportunity to recover from preconditioning stress. The experimental design is shown in Fig. 5.

### Islet characterisation

Subsequent to islet purification, HP and post culture in hypoxic atmosphere, islet yield was quantified as islet particle number (IN) and converted to islet equivalents (IEQ) as previously described in detail^76^. Counting and size categorisation of islets stained with dithizone (Sigma-Aldrich, Dorset, U.K.) was performed by two independent persons in a non-blinded fashion using a 50 µm-grid implemented in the ocular of an inverted microscope (Leica Microsystems Limited, MIlton Keynes, U.K.). Islet morphological integrity was determined calculating the islet fragmentation index, which was calculated as the ratio of IN over IEQ^75^. Islet viability was assessed utilising 0.67 µmol/L of fluorescein diacetate (FDA, Sigma-Aldrich) and 4.0 µmol/L of propidium iodide (PI, Sigma-Aldrich) for staining of viable and dead cells, respectively^77^. The fluorescence intensity (FI) of FDA-PI was quantified in duplicate samples utilising a automated procedure by means of a fluorometric plate reader as previously described^30^. In order to provide an integrative parameter that considers the recovery of living islet cells only which had been exclusively stained by FDA and had not being penetrated by PI, we established islet overall survival. Overall survival was calculated by multiplying the proportion of living cells with the proportional recovery of initially incubated islets. As the initial islet aliquot yield is 100%, overall survival calculated pretreatment is identical with islet viability at that time point.

In vitro function of 10 hand-picked islets with an average diameter of 150–200 µm in diameter was assessed in duplicate during static glucose incubation. Islets were seeded on 0.8 µm-pore size filter inserts, transferred into 24-well plates and sequentially incubated for 45 min in 1 mL of bicarbonate-free CMRL 1066 (Applichem, Darmstadt, Germany) supplemented with 2.0 mmol/L glucose followed by 45 min at 20 mmol/L and finally re-incubated for a second period of 45 min at 2 mmol/L glucose. After glucose stimulation, islets were recovered and sonified in distilled water prior to insulin extraction in acid ethanol. Intracellular and secreted insulin was determined utilizing an enzyme immunoassay specific for rat insulin (Mercodia, Uppsala, Sweden). The glucose stimulation index (SI) was calculated by dividing the insulin release at 20 mmol/L glucose by the mean of the two basal periods.

Production of reactive oxygen species (ROS) was determined by measuring the intra-islet conversion of dichlorofluorescein diacetate (DCFA-DA, Sigma-Aldrich) into fluorescent dichlorodihydrofluorescein (DCFH) by means of a fluorometric plate reader as previously described in detail^34^. DNA fragmentation was validated using the Cell Death Detection ELISA kit according to its manual (Roche Diagnostic GmbH, Mannheim, Germany). Early apoptosis was measured by simultaneous staining with Annexin-V FITC (Becton Dickinson Biosciences, Oxford, United Kingdom) and PI used at a concentration of 450 ng/mL and 4.0 µmol/L, respectively.

### Quantitative real-time polymerase chain reaction

Gene expression of cultured islets (n = 7) was measured using Taqman-based quantitative real-time polymerase chain reaction (qRT-PCR). Briefly, total RNA was extracted from 100 cultured handpicked islets of similar size (150–200 µm) using the RNeasy Micro kit (Qiagen, Germany) before being run in triplicate for 35 cycles on a QuantStudio 7 (Applied Biosystems, CA, USA) using the CellsDirect One-Step qRT-PCR kit (Invitrogen, CA, USA). Duplex reactions were performed using TaqMan assays specific for the target genes BCL-2 associated X protein (BAX, Rn01480161_g1), C/enhancer-binding protein homologous protein (CHOP) (CHOP, Rn00492098_g1), heme oxygenase-1 (HO-1) (HMOX1, Rn00561387_m1), hypoxia-inducible factor-1α (HIF-1α) (HIF1A, Rn01472831_m1), vascular endothelial growth factor A (VEGF-A) (VEGFA Rn01511602_m1), all normalized to 18S ribosomal RNA (rRNA) (18S rRNA, Rn01452893_g1). All primers were provided by Applied Biosystems (Warrington, U.K.). Quantitative values were obtained using the threshold cycle number and the x-fold change in expression using the ΔΔC_T_ method^78^.

### Statistical analysis

Statistical analysis and graphical presentations were performed utilizing Prism 9.1.1 (GraphPad, La Jolla, USA; www.graphpad.com). Analysis of data was carried out by the nonparametric Friedman test followed by Dunn’s test for multiple comparisons or by the Wilcoxon test for subsequent insulin release at 2 and 20 mmol/L of glucose. Comparisons between corresponding parameters collected in HBD and NHBD were done using the Mann–Whitney test. Correlation analysis was performed calculating nonparametric Spearman’s correlation coefficient (r). Differences were considered significant at *p* less than 0.05. *P*-values larger than 0.05 were termed nonsignificant (NS). Results are expressed as mean ± standard error (SEM). If appropriate, data were normalised to data determined pretreatment or to sham-treated islets.

## Data Availability

Data generated in the current study are available from the corresponding author upon reasonable request.
